# OA09.01. Massage therapy for osteoarthritis of the knee: a randomized dose-finding trial

**DOI:** 10.1186/1472-6882-12-S1-O33

**Published:** 2012-06-12

**Authors:** A Perlman, A Ali, V Njike, D Hom, A Davidi, S Gould-Fogerite, C Milak, D Katz

**Affiliations:** 1 Duke University, Durham, USA; 2 Yale University School of Medicine, Prevention Research Center, Derby, USA; 3 Boston Medical Center, Boston, USA; 4 University of Medicine and Dentistry of New Jersey, Newark, USA

## Purpose

In a previous trial of massage for osteoarthritis (OA) of the knee, we demonstrated feasibility, safety and possible efficacy, with benefits that persisted at least 8 weeks beyond treatment termination.

## Methods

We performed a RCT to identify the optimal dose of massage within an 8-week treatment regimen and to further examine durability of response. Participants were 125 adults with OA of the knee, randomized to one of four 8-week regimens of a standardized Swedish massage regimen (30 or 60 min weekly or biweekly) or to a Usual Care control. Outcomes included the Western Ontario and McMaster Universities Arthritis Index (WOMAC), visual analog pain scale, range of motion, and time to walk 50 feet, assessed at baseline, 8-, 16-, and 24-weeks.

## Results

WOMAC Global scores improved significantly (24.0 points, 95% CI ranged from 15.3-32.7) in the 60-minute massage groups compared to Usual Care (6.3 points, 95% CI 0.1-12.8) at the primary endpoint of 8-weeks. WOMAC subscales of pain and functionality, as well as the visual analog pain scale also demonstrated significant improvements in the 60-minute doses compared to usual care. No significant differences were seen in range of motion at 8-weeks, and no significant effects were seen in any outcome measure at 24-weeks compared to usual care. A dose-response curve based on WOMAC Global scores shows increasing effect with greater total time of massage, but with a plateau at the 60-minute/week dose.

## Conclusion

Given the superior convenience of a once-weekly protocol, cost savings, and consistency with a typical real-world massage protocol, the 60-minute once weekly dose was determined to be optimal, establishing a standard for future trials.

